# Antimicrobial Stewardship: Development and Pilot of an Organisational Peer-to-Peer Review Tool to Improve Service Provision in Line with National Guidance

**DOI:** 10.3390/antibiotics10010044

**Published:** 2021-01-05

**Authors:** Olaolu Oloyede, Emma Cramp, Diane Ashiru-Oredope

**Affiliations:** 1 Public Health England, London SE1 8UG, UK; Olaolu.oloyede@phe.gov.uk; 2 Patient Safety, NHS Improvement, London SE1 6LH, UK; emma.cramp@nhs.net

**Keywords:** AMS, antimicrobial resistance, antimicrobial stewardship intervention, PDSA cycle

## Abstract

Antimicrobial resistance continues to be a considerable threat to global public health due to the persistent inappropriate use of antibiotics. Antimicrobial stewardship (AMS) programs are essential in reducing the growth and spread of antibiotic resistance, in an environment which lacks incentives for the development of new antibiotics. Over the years, a variety of resources have been developed to strengthen antimicrobial stewardship. However, the differences in resources available present a challenge for organisations/teams to establish the best resources to utilise for service provision. A peer review tool was formulated using four national documents on AMS and tested through three phases with feedback. A survey method was used to collect feedback on the validity, feasibility, and impact of the AMS peer review tool. Feedback received was positive from the earlier pilots. The tool was found to be useful at identifying areas of good practice and gaps in antimicrobial stewardship across various pilot sites. Feedback suggests the tool is useful for promoting improvements to AMS programs and highlights that the content and features of the tool are appropriate for evaluating stewardship.

## 1. Introduction

Antimicrobial stewardship (AMS) is an organisational or healthcare system-wide approach to improving and optimising antimicrobial therapy through the promotion and monitoring of the appropriate use of antimicrobials to prevent the development of resistance. Evidence suggests that a coordinated and comprehensive AMS programme is vital in tackling the emergence of antimicrobial resistance [1].

Several national AMS guidance and toolkits have been developed to support and encourage best practice in acute National Health Service (NHS) hospitals in England, as well as the goals outlined in the UK 5-year National Action Plan 2019–2024. These collections of resources, produced by different expert groups and at different time points, present a challenge for organisations/teams to establish the best resources to improve service provision. In response to this, the consolidation of recommendations from these national resources into one complete AMS tool can provide clarity to enable adherence to national guidance, thus consistent and better stewardship. The Health Foundation defines peer review as the professional assessment against standards of the organisation on healthcare processes and quality of work, to foster improvement [2]. The peer review tool developed aims to support hospitals to systematically review their processes for appropriate antimicrobial prescribing, stewardship and improving patient outcomes. The tool is intended for use at the host site with an external peer reviewer, to allow for an impartial assessment of AMS practices and development of an improvement plan [3].

Organisational peer-to-peer reviews offer an objective assessment to drive internal improvement through the evaluation of a provider by another organisation without the need for formal regulatory authority involvement. Cases of organisational peer-to-peer review are rare in healthcare; an example of this approach includes the UK National Chronic Obstructive Pulmonary Disease (COPD) Resources and Outcomes Project and the regional intervention to improve the hospital mortality associated with coronary artery bypass graft surgery (The Northern New England Cardiovascular Disease Study Group) [4].

To prevent the growing issue of antimicrobial resistance (AMR), NHS England (now NHS England & Improvement) launched the world’s largest healthcare incentive scheme for hospitals and other health service providers. The programmed offered NHS Trusts incentive funding valued up to £150 million to support expert clinicians and pharmacist’s assessment and reduction of inappropriate antimicrobial prescription [5]. The development of the AMS peer review tool with a plan-do-study-act (PDSA) cycle approach aims to support national investment in tackling AMR issues through organisational peer-to-peer reviews.

## 2. Results

PDSA Cycle 1:

In 2016, positive feedback was received from all participants from the East of England pilot using the first version of the tool [6]. The tool was found to be beneficial at identifying areas of good practice and gaps in antimicrobial stewardship at each pilot site, as well as presenting opportunities to learn from peers. The participants found that the tool was relatively easy to use and indicated peer review visits annually would be adequate.

The average length of time to undertake the peer review was five hours in total. These five hours where made up of approximately 2 h for reviewing necessary documents prior to visiting the host organisation and 3 h to conduct the site visit which included attendance at the AMS committee meeting and visiting a ward area to interview healthcare workers.

PDSA Cycle 2:

In 2018, following the presentation of the peer review tool to the national multidisciplinary group on antimicrobial resistance and utilisation, the English surveillance programme for antimicrobial utilisation and resistance (ESPAUR) group; the number of indicators in the tool was reduced from 101 to 37 following a two stage process and updated to include indicators from the current antimicrobial stewardship guidance and toolkits. Similar indicators were merged so that repetition was minimised, and themes were grouped together. Table 1 summarises the number of indicators from each stage of the toolkit development during cycle 2.

PDSA Cycle 3:

In 2019, feedback on the revised shortened tool from another pilot of five participating acute hospitals (three teaching and two non-teaching trusts) in two regions of England suggested a two-week lead time for submission of the hospitals’ documented evidence of the AMS programme was appropriate and the documents shared were found to be “mostly relevant”. It was also viewed that the tool could be beneficial in “promoting shared learning across the hospital stewardship programmes” and the peer review should be repeated every three years. One of the challenges highlighted was arranging a suitable date for the peer review on-site visit for both parties.

Despite the revision of the tool and reduction in the number of questions, the time taken to complete the peer review did not decrease from the initial pilot, with an average of 5 h (1.6 h to review the document before the site visit and 3.4 h for the on-site visit). The reason for this was because although the indicators were merged to form fewer questions for the reviewer, the themes required an in-depth review which did not change the overall amount of time needed for the review. In circumstances where the NHS Trust’s AMS programme is satisfactory, the tool was considered time-consuming, but the outcome of the visit provided assurance for the AMS team. All participants agreed that the benefit from the tool included its application to reinforce good practice and benchmarking against peers.

The majority of respondents highlighted that all domains assessing the NHS Trust’s antimicrobial stewardship programme were either “very relevant” or “relevant” with the exception of the “Patient and Carers” domain in which some responded “neutral”. The consensus was that the tool would be best used by healthcare professionals with an infection specialist background, as well as an excellent resource to promote shared learning across the hospital stewardship programme. Some of the planned actions by the host organisations following the peer review were:

- Review of the antimicrobial team at a future ASC meeting,
- Analysis of detailed data at consultant and ward level data,
- To re-visit the area highlighted that require strengthening,
- Develop an education plan, and
- Develop audit and feedback plan.

## 3. Discussion

The development of the AMS peer review tool focussed on establishing a resource that amalgamates the variety of national guidance and tools on good antimicrobial stewardship into a single resource. Thus, providing a comprehensive and structured instrument to strengthen an NHS Trust antimicrobial stewardship programme. However, selecting a sample of acute NHS Trusts conveniently located within the same geography to pilot the tool at various stages of development proved to be quite challenging. With the variation across NHS Trusts with different processes, cultures, capacity, and attitudes on tackling antimicrobial resistance, there is a need to broaden the geographical spread sample that will provide a more detailed insight to the feasibility of the tool. Thus, it was intended that the tool would be piloted across at least two hospital trusts in all the NHS regions to have a representative sample in the development of the tool. However, having a broad and large sample of secondary care institutions (National Health Service Trusts) willing to participate in the pilot proved to be a significant challenge as coordinating the on-site visit between both organisations was dependent on the dates the antimicrobial stewardship committee meetings were due to take place, as well as the pharmacist’s clinical commitments, and annual leave. During the pilot, it was highlighted that having a central team to coordinate organisation of on-site visit across the hospitals is important and beneficial; however, this strategy will require additional resources within each regional health system. To support widespread uptake of such measuring instruments and opportunity for regional or national perspective on the variation of stewardship programmes, implementation of future organisational peer-to-peer review tools could benefit from a central resource to coordinate dates and visits to reduce burden on individual teams.

The feedback from the pilot showed that participants found the tool to be a useful resource that encourages shared learning between peers and identifying gaps within their antimicrobial stewardship programme. However, the results of the pilots may be biased towards individuals with a keen specialist interest in improvement measuresand actively involved in promoting and implementing good stewardship practices across the hospital. Furthermore, there is the potential for selection bias from the pilots, as there was an overrepresentation of pharmacists conducting the peer review (all reviews completed by pharmacists). Although it is hoped that through the feedback from the ESPAUR oversight group (which includes members from a wide range of backgrounds such as microbiology consultants, members of the dental profession, and nurses) who provided critical review of the tool, some of the selection bias may have been addressed. The use of the tool is not intended to be exclusive to a specific profession, but rather to be used by any healthcare professional with an antimicrobial stewarship/infection management background.

In addition, the evidence across various medical areas suggests that following a peer review, improvements to services can sometimes occur slowly with inconsistent outcomes. It has been suggested, to achieve better results that will deliver change and improved services, that a multidisciplinary peer review visit may be a more attractive mechanism as a collective and agreed strategy to implement the recommendations is likely to breed success [7]. Organisation-wide support on antimicrobial stewardship is considered crucial to addressing AMR, thus without this, minimal impact may be expected following visits.

The iterations of developing the tool demonstrated that the content and elements of the assessment tool are suitable for evaluating stewardship, thus providing a robust and systematic approach. The challenge from the outset was coordinating a system-wide simultaneous uptake within regions of the AMS peer review tool, with a proposed annual visit to reassess and support consistent improvement in stewardship programmes.

## 4. Materials and Methods

The AMS peer review tool was originally developed and piloted by East of England Antimicrobial Pharmacists Network in 2016 across eight NHS acute Trusts. The tool includes consolidated recommendations from a number of national AMS guidance and toolkits [8,9,10,11] into one easy-to-use document, assessing an organisation’s stewardship programme on the following domains:

- AMS leadership and management,
- Antimicrobial prescribing management,
- Surveillance, resistance and standards,
- Risk assessments for antimicrobials,
- Patient and carers, and
- Education and training on the use of antimicrobials

The intention was to develop a voluntary tool (available to download as a Supplementary Material) to strengthen antimicrobial stewardship in acute hospitals through the facilitation of organisational peer reviews within the regional health systems in England. The tool was tested through three phases with the feedback and outcome shared with the English surveillance programme for antimicrobial utilisation and resistance (ESPAUR) group, consisting of experts from various backgrounds (microbiologist, paediatricians, infection disease specialist, and pharmacists). Through the phases of developing the AMS peer review tool, a survey method was used to collect feedback on the validity, feasibility, and impact.

The findings of the pilot were first presented to ESPAUR oversight group in January 2017. The average length of time to perform a peer review was five hours (two hours to review key documents before the site visit and three hours to carry out the site visit which included attending an AMS committee meeting). Feedback from the ESPAUR members was to simplify the tool by reducing the number of indicators before the next piloting stage. The tool was further updated in January 2019 and validated using the checklist outlined by Pulcini et al. [12] in their publication on developing a global checklist for hospital AMS programmes. The updated version of the tool was shared via the AMR network leads with antimicrobial pharmacists across some acute hospitals in England to request their participation to pilot. Incorporated into the updated peer-review process was a pre-visit stage, to encourage more focussed and productive on-site visits. Volunteering hospitals were paired within their region to avoid long-distance travel to conduct the peer-review.

The three iterations of testing and evaluating feedback were used to validate and refine the tool, with time to completion measured, and participants’ experience of utilising the tool collected (Table 2).

### Using the AMS Peer Review Tool

It was recommended that those wishing to pilot the tool (available as supplementary material) consider choosing peer hospitals based on geographical location to minimise the amount of travel time, and that the whole review process may take one full working day to complete and that peer review process can be considered every two to three years (as this allows enough time to lapse to accrue the benefits of the peer review and appropriate time for reassessment), or more frequently if an improvement plan is implemented. The peer review may be carried out by an individual or team from an external organisation, which is not limited to the list below, and may include one or more of the following:

- Antimicrobial pharmacist
- Infection prevention control/AMS Nurse
- Director (lead) of infection prevention and control (DIPC)
- Commissioner
- Clinical microbiologist or ID physician
- Other member of AMS committee
- National or regional antimicrobial stewardship leads/committee members

The AMS peer review tool outlines key aspects across the six AMS domains mentioned earlier to be critically reviewed and assessed by the host organisation and peer reviewer. The recommended process for conducting the peer review is outlined below:

Step 1

Plan and schedule the onsite visit to occur ideally on the day the Antimicrobial Stewardship Committee (ASC) is held. This would allow the reviewer to witness first-hand the attendance, management, and leadership at the meetings. The host organisation should schedule an opportunity for the peer review team to meet with senior clinicians and managers. The date and time of visit should be scheduled during less busy periods and where possible consider staff availability due to annual leave.

Step 2

Host organisation to prepare documents listed in the tool for submission to peer reviewer two weeks ahead of scheduled visit and self-assess AMS prior to peer reviewer visit.

Peer reviewer to review documents submitted by host organisation and prepare approach prior to visit.

Step 3

During the onsite visit, the reviewers should speak with clinical staff on the ward. In addition, where it is considered necessary or additional benefit for the review process, reviewers may also consider having discussions with senior clinicians, and managers including the medical director, lead for infection prevention and control (e.g., the director for IPC in the UK (DIPC)), director of nursing, microbiologist lead for AMS and chief pharmacist within the NHS Trust, the director of nursing and quality, AMR/AMS lead pharmacist and chief pharmacist within the CCG, and the system AMR lead.

Step 4

Peer review report to be submitted within the agreed time frame at the onsite visit. The report should outline the areas of success and opportunities for improvement.

## 5. Conclusions

The pilots were important in assessing the feasibility of the tool and outlining the barriers for use. The feedback from participants and expert group suggest that the tool is best used where a gap or issue has been identified within a hospital stewardship programme, with the tool providing a comprehensive review to help develop strategies for improvements. The tool also presents an opportunity for regional antimicrobial groups to voluntarily lead on coordinating the visits to share best practices amongst hospital within the region. Overall, this quality improvement project showed there was a need for tools that support organisational peer-to-peer review. However, future work on developing such a quality improvement model need to build in considerations that will reduce the time burden and pressure for the reviewers.

## Figures and Tables

**Table 1:** Number of indicators at each stage of the toolkit development.

Section	Area	Original Tool	Stage 1	Stage 2
**1**	AMS management team/antimicrobial stewardship committee (ASC)	15	10	6
**2**	AM Prescribing management	48	20	18
**3**	Surveillance, resistance, and standards	12	7	1
**4**	Risk assessment for antimicrobials	7	7	5
**5**	Patients and carers	5	5	4
**6**	Education and training on the use of antimicrobials	6	4	3
**-**	Antimicrobial pharmacist	8	6	Moved *
	Total number of indicators	101	59	37

* Moved to the AMS management team/ASC.

**Table 2:** Summary of the plan-do-study-act (PDSA) cycles.

Cycle	Completed by:	Intervention
**1**	January 2016	Pilot of original tool and feedback by eight organisations within one region of England through the East of England Antimicrobial Pharmacists Network.
	January 2017	Results of pilot presented to the English surveillance programme for antimicrobial utilisation and resistance (ESPAUR).
**2**	March 2018	Tool updated in line with latest national guidance and toolkits.
	July 2018	Updated tool including indicators from national guidance and toolkits presented to EPSAUR.
	December 2018	Two stages of review and feedback to reduce number of indicators assessed through the tool.
	January 2019	Shortened version presented to ESPAUR group.
**3**	April/May 2019	Pilot of updated peer review tool with five NHS Trusts across 2 regions.
	July 2019	Pilot output presented to ESPAUR and final tool endorsed with recommendations methods for cascade.

## Data Availability

Data is contained within the article or supplementary material.

## Supplementary Materials

The following are available online at https://www.mdpi.com/2079-6382/10/1/44/s1, Antimicrobial Stewardship (AMS) Peer Review Tool as PDF and editable excel document.
