# Supplementary material for: Antimicrobial Stewardship: Development and Pilot of an Organisational Peer-to-Peer Review Tool to Improve Service Provision in Line with National Guidance
Source: Antibiotics (Basel). 2021 Jan 5;10(1):44. doi: 10.3390/antibiotics10010044 (PMC7824785; doi:10.3390/antibiotics10010044)
Supplement: Supplementary file 1 [file Oloyede et al_AMS Peer Review Tool.pdf › Oloyede et al_AMS Peer Review Tool.pdf]

Antimicrobial Stewardship (AMS) Peer Review Tool

Originally developed by East of England Antimicrobial Pharmacy Network, 2016
Updated by PHE English Surveillance for Antimicrobial Utilisation and Resistance Group, 2020

Hospital: ______________________ Date ______________________

Host AMS practitioner / designation: ______________________

Reviewer / designation: ______________________

Introduction

The aim of the AMS Peer Review Tool is to support hospitals to undertake a complete review of their antimicrobial stewardship programme. It is well documented that robust systems and processes for antimicrobial use are needed to address the threat of antimicrobial resistance, thus effective strategies that incentivise clinicians and organisations to periodically assess against professional standards to improve quality of care is beneficial. This tool will serve as an opportunity for external reviewers to assess the strengths and weaknesses of AMS programmes and highlight areas for improvement for the host site.

Organisational peer-to-peer reviews offer an objective assessment to drive internal improvement, through the evaluation of a provider by another organisation without the need of formal regulatory authority involvement(1). Examples of this approach are the UK National Chronic Obstructive Pulmonary Disease Resources and Outcomes Project(2) and the regional intervention to improve the hospital mortality associated with coronary artery bypass graft surgery(3).

The initial version of this tool was developed by the East of England Antimicrobial Pharmacy Infection Network in March 2016 and has since been updated in line with current guidance. It has been created using guidance from a number of resources including Public Health England (PHE) Start Smart then Focus: Antimicrobial stewardship toolkit for English Hospitals, National Institute for Health and Care Excellence (NICE) guideline Antimicrobial stewardship: systems and processes for effective antimicrobial medicine use, Antimicrobial Self-Assessment Toolkit (ASAT) for acute hospitals and the Health and Social Care Act 2008: Code of Practice. It has also been adapted from The Healthcare Environment Inspectorate antimicrobial Inspection Tool produced by Healthcare Improvement Scotland.

Originally developed by East of England Antimicrobial Pharmacy Network, 2016
Updated by PHE English Surveillance for Antimicrobial Utilisation and Resistance Group, 2020

This toolkit is intended to be used for an antimicrobial stewardship peer review in acute hospitals. It is recommended that the host antimicrobial team complete this tool prior to the peer review visit in order to minimise the length of time the process takes. The reviewer will review the evidence (from an initial pilot this may take up to 3 hours) submitted to the reviewer in advance (at least two weeks before the peer review) by a member of the host antimicrobial stewardship team (AMT) (please see a recommended list of documents below).. The reviewer will also need to speak to a number of different types of clinical staff on a ward of their choice to determine if clinical staff members are aware of how to access antimicrobial guidelines and policies.

Peer hospitals should be chosen based on geographical location to minimise the amount of travel time. The whole review process may take one full working day to complete. A peer review process can be considered every two years.

Peer reviewer(s)

The peer review may be carried out by an individual or team from an external organisation, which may include one or more of the following:

Antimicrobial Pharmacist
Infection Prevention Control/AMS Nurse
Commissioner
Clinical Microbiologist or ID Physician
Other Member of AMS committee

Preparation of peer review

Host organisation

- Submit required documents at least 2 weeks prior to visit.
    - Collate documents listed in the table below to be emailed in advance to the peer reviewer to inform them about any specific area that requires additional attention on visit.
    - Complete and submit trust antimicrobial prescribing and resistance data over the preceding 12 months to peer review (appendix 2).
- Ideally the reviewer should attend the host site on a day when the Antimicrobial Stewardship Committee (ASC) is held so that they can witness first-hand the attendance, management and leadership at the meetings.

Peer reviewer(s)

- Spend time reviewing documents and data submitted prior to visit

- Prepare approach and if going as part of a team agree the roles for each member of the team during the visit.

**Suggested documents to be sent to reviewer at least two weeks prior to peer review (tick documents shared):**
☐ Empirical Antibiotic Guidelines for each clinical speciality } or direct reviewer to smartphone guideline app ☐<br>☐ Antibiotic prescribing policy<br>☐ Terms of reference (TOR) for Antimicrobial Stewardship Committee (ASC)<br>☐ Minutes from Antimicrobial Stewardship Committee<br>☐ Strategy for Antimicrobial Stewardship (AMS)<br>☐ Infection Prevention and Control strategy<br>☐ Job Description for Director of Infection Prevention and Control (DIPC)<br>☐ Trust Board annual report<br>☐ Attendance tracker for Antimicrobial Stewardship Committee<br>☐ Restricted Antibiotic List<br>☐ Work plan for AMS<br>☐ Audit plan for AMS<br>☐ Education and training strategy for AMS<br>☐ Copy of the antimicrobial formulary<br>☐ Audit data feedback report<br>☐ Presentation slides for education and training sessions<br>☐ Completed NICE AMS baseline assessment tool - https://www.nice.org.uk/guidance/ng15/resources<br>☐ Healthcare Associated Infection action plan<br>☐ Evidence of AMS quality improvement initiative<br>☐ Other documents - please state:

Key:

ToR = Terms of reference

ASC = Antimicrobial stewardship committee

NICE = National Institute for Health and Care Excellence

SSTF = Start Smart then Focus

ASAT = Antimicrobial Self-Assessment Tool
AMP = Antimicrobial Pharmacist

Antimicrobial Prescribing and Resistance Outcome Measures

**Submit trust antimicrobial prescribing and resistance data for review** (tick shared data *):
☐ Four quarter rolling rate of total antibiotic prescribing per 1000 admissions; (Source – Fingertips)<br>☐ Four quarter rolling rate of carbapenem prescribing per 1000 admissions; (Source – Fingertips)<br>☐ Proportion of total antibiotic prescribing from the "Access" category of the WHO Essential Medicines List AWaRe index; (Source – Fingertips)<br>☐ Defined daily dose of antibiotics dispensed by Acute Trusts pharmacies to all inpatients and outpatients per 1000 admissions; (Source – Fingertips)<br>☐ Defined daily dose of carbapenems dispensed by Acute Trusts pharmacies to all inpatients and outpatients per 1000 admissions; (Source – Fingertips)<br>☐ Percentage of antibiotic prescriptions with evidence of review within 72 hours; by quarter (Source – Fingertips)<br>☐ C. difficile hospital-onset rates by reporting acute Trust and financial year (Source – Fingertips, Internal data)<br>☐ C. difficile infection counts and 12-month rolling rates of all cases, by reporting acute trust and month (Source – Fingertips, Internal data)<br>☐ E. coli bacteraemia hospital-onset counts and rates by NHS acute trust and financial year (Source – Fingertips, DCS)<br>☐ E. coli bacteraemia cases counts and 12-month rolling rates, by reporting acute trust and month (Source – Fingertips, DCS)<br>☐ Percentage of frontline healthcare workers vaccinated with the seasonal influenza vaccine by NHS Acute Trust (Source – Fingertips, OH internal data)

*Latest data required along with a line graph to assess the trend

Originally developed by East of England Antimicrobial Pharmacy Network, 2016
Updated by PHE English Surveillance for Antimicrobial Utilisation and Resistance Group, 2020

1. Antimicrobial Stewardship Leadership and Management

No.	Criteria		Self-assessment	Evidence Provided	Reviewers Assessment
1.1	Has the hospital management (board of directors, divisional directors, medical director, DIPC etc.) formally identified antimicrobial stewardship as a priority objective for the institution and included it in its key performance indicators?				
1.2	Is there dedicated, sustainable and sufficient budgeted financial support for antimicrobial stewardship activities, training or IT support?<br>*(e.g. support for salary, information technology)*				
1.3	Does the trust have a **robust** governance structure for the AMS programme/committee?<br>*a. Is there an Antimicrobial Stewardship committee?*<br>*b. Does the committee have a Terms of Reference?*<br>*c. Is there a MDT core membership with quorate list within the ToR?*<br>*d. Does the committee meet more than quarterly?*<br>*e. Is there a written AMS strategy/action plan that monitors adherence to good AMS prescribing principles in all clinical areas (at least annually)?*<br>*f. Actions are identified to address non-compliance with local guidance or unexpected trends in prescribing. These actions should be documented within the minutes of the ASC. Repeated non-compliance is formally investigated and is challenged by DIPC.*				

Originally developed by East of England Antimicrobial Pharmacy Network, 2016
Updated by PHE English Surveillance for Antimicrobial Utilisation and Resistance Group, 2020

No.	Criteria		Self-assessment	Evidence Provided	Reviewers Assessment
	g. *Are there clear lines (formal/written) of accountability to a higher committee e.g. Drug and Therapeutics Committee, Clinical Governance Committee or Infection Control Committee? Minutes from ASC are discussed at this higher level meeting?*<br>h. *Is a dedicated report produced at least annually which includes e.g. antibiotic use trends and/or prescription improvement initiatives, with time committed short term and long term measurable goals/targets for optimising antibiotic use?*				
1.4	Is there senior management support/responsibility for AMS?<br>a. *Does the Director of Infection Prevention and Control (DIPC) have AMS incorporated into their Job Description*<br>b. *Does the Trust/hospital have a senior manager responsible for AMS activities?*<br>c. *Is AMS addressed within the Trust Infection Prevention and Control (IPC) Strategy?*				
1.5	Who is part of the ASC? Has a whole health economy approach been adopted?<br>☐ Antimicrobial Pharmacist<br>☐ Microbiologist<br>☐ Infectious Diseases consultant<br>☐ Senior Nurse<br>☐ Paediatrician<br>☐ Primary Care Representative<br>☐ Acute Care Physician<br>☐ Senior member of the Pharmacy Management Team				

Originally developed by East of England Antimicrobial Pharmacy Network, 2016
Updated by PHE English Surveillance for Antimicrobial Utilisation and Resistance Group, 2020

No.	Criteria		Self-assessment	Evidence Provided	Reviewers Assessment
	☐ Anaesthetist<br>☐ Surgeon<br>☐ Director of Infection Prevention and Control<br>☐ Infection Prevention and Control Nurse<br>☐ Other Pharmacist<br>☐ Infection Prevention and Control Consultant<br>☐ CCG/CSU Pharmacist<br>☐ CCG Infection Prevention and Control Lead<br>☐ CCG commissioner<br>☐ Sepsis Lead Consultant<br>☐ Critical care outreach team nurse<br>☐ Lead Consultant for acute medicine<br>☐ Other (please specify below)				
1.6	Is there an Antimicrobial Pharmacist (AMP) identified as a leader for antimicrobial stewardship activities at your hospital?<br>a. *Does the AMP have written objectives and a PDP relating to the AMS strategy*<br>b. *Reviewing outpatient parenteral antibiotic therapy patients*<br>c. *The AMP plays an active part in local AMS networks across all care settings (i.e. local AMS networks, regional networks, UKCPA member)*<br>d. *Does the lead AMP have specialist training in infection (i.e. MSc in Infection Management) or greater than 3 years' experience within the specialist role?*<br>e. *Is the lead antimicrobial pharmacist working towards RPS faculty submission?*<br>f. *What whole time equivalent (WTE) AMP/500 beds are spent on AM duties?*				

2. Antimicrobial Prescribing Management

No.	Criteria		Self-assessment	Evidence Provided	Reviewers Assessment
**Policy**					
2.1	Is there a robust AMS policy that clearly states the overall principles of AM use?<br>*(i.e. Antimicrobial prescribing policy which outlines responsibilities for staff)*				
2.2	Does the policy clearly define roles, responsibility and procedures of the antimicrobial team and antimicrobial use?<br>*a. initiation of AM within one hour for sepsis*<br>*b. narrow spectrum, reviewing switching formulation of antibiotics*<br>*c. reviewing of microbiology results within 48-72 hours*<br>*d. prescribers documenting the review, use and switching of IV AMs*<br>e. *reminder for prescriber to consider resistant pathogens*				
2.3	Does the policy or guidelines include the Start Smart then Focus treatment algorithm?				
**Guidelines**					
2.4	Do you have a robust method of sharing guidelines?				
2.5	Is there an up to date AM formulary?<br>*(i.e. a list of antimicrobials that have been approved for use in a hospital, specifying whether the drugs are unrestricted, restricted (approval of*				

Originally developed by East of England Antimicrobial Pharmacy Network, 2016
Updated by PHE English Surveillance for Antimicrobial Utilisation and Resistance Group, 2020

No.	Criteria		Self-assessment	Evidence Provided	Reviewers Assessment
	an antimicrobial stewardship team member is required) or permitted for specific conditions)				
2.6	Is there a system for control of entry onto the formulary for new AMs and ongoing monitoring of costs, prescribing and patient outcomes?				
2.7	Is selection for the guidelines informed by local sensitivity data?				
**Clinical Management**					
2.8	Does your hospital have available and up-to-date recommendations (updated at least every year or when a new guideline is published) for AM guideline *(diagnosis, prevention and treatment)*, based on international/national evidence-based guidelines (such as NICE common infection guidelines) and local susceptibility (when possible), to assist with antimicrobial selection *(indication, agent, dose, route, duration)* for common clinical conditions, developed with the involvement of a clinical microbiologist or lead practitioner?				
2.9	Has an MDT quality improvement programme for AMS been developed and is this sustained?				
2.10	Does your hospital support the antimicrobial stewardship activities/ strategy with adequate information technology services? *(e.g. electronic summaries and guidelines available to wards, smartphone apps, decision support software)*				
**Microbiology**					
2.11	Does the Microbiology lab use selective reporting of results in line with formulary choices?				

Originally developed by East of England Antimicrobial Pharmacy Network, 2016
Updated by PHE English Surveillance for Antimicrobial Utilisation and Resistance Group, 2020

No.	Criteria			Self-assessment			Evidence Provided	Reviewers Assessment
2.12	Are there timely microbiology results (including susceptibility data) preferably within 48 hours?							
2.13	Is susceptibility data (drug-bug combinations) and antimicrobial consumption reported to the national surveillance body?							
2.14	Is advice from a microbiologist/ID physician available by phone 24 hours a day?							
**Ward Round & Stewardship**								
2.15	What ward rounds take place, what departments, frequency, and who attends?	Wards	Frequency	Microbiologist	IPC Nurse	Pharmacist		
	Clostridium difficile							
	General AMS ward round							
	Intensive Care Unit							
	Respiratory disease							
	Areas of high prescribing							
**Feedback**								
2.16	Is there evidence of audits/review that align with national AMR strategy/targets (e.g. CQUINs, 48-72hours reviews) and adherence to local guidelines?<br>a. Is the advice and data easily accessible?							

No.	Criteria		Self-assessment	Evidence Provided	Reviewers Assessment
	b. Are the outcomes communicated to all staff in particular those who prescribe antibiotics?				
2.17	Is evidence of an antimicrobial (AM) review/audit of specific antimicrobial agents or clinical conditions that can is easily accessed?<br>*(to be shared with the Trust board and other members of staff)*				
2.18	Is the information on AM prescribing feedback to prescribers?<br>*a. Is there regular feedback of audit data at a directorate level*<br>*b. Is there a system in place to feedback to prescribers patient safety incidents related to AM use and*<br>c. *Is there a system reporting lack of compliance to guidelines*				
2.19	Are local systems and processes are in place for encouraging peer review of prescribing AMs?				

Originally developed by East of England Antimicrobial Pharmacy Network, 2016
Updated by PHE English Surveillance for Antimicrobial Utilisation and Resistance Group, 2020

3. Surveillance, Resistance and Standards

No.	Criteria		Self-assessment	Evidence Provided	Reviewers Assessment
3.1	Does your hospital monitor and report antimicrobial consumption and prescribing data?<br>a. *Broad spectrum antibiotics consumption*<br>b. *Local resistance patterns*<br>c. *Patient outcomes (e.g. LOS, mortality rates & readmissions)*<br>d. *Use national surveillance data for benchmarking*<br>e. *Measured ward level and consultant level*				

Originally developed by East of England Antimicrobial Pharmacy Network, 2016
Updated by PHE English Surveillance for Antimicrobial Utilisation and Resistance Group, 2020

4. Risk Assessment for Antimicrobials

No.	Criteria		Self-assessment	Evidence Provided	Reviewers Assessment
4.1	Do you have the following guidance to improve patient safety?<br>*a. AM allergies guidance*<br>*b. IV to oral switch*<br>c. *Therapeutic Drug Monitoring guidance*				
4.2	Are incidents involving AMs reviewed at ASC meetings and incorporated into patient safety reporting systems to Boards and Commissioners? *(particularly hospital admissions for potentially avoidable life threatening infections, CDI and anaphylaxis)*				
4.3	Systems and processes are in place for identifying and reviewing whether hospital admissions are linked to previous prescribing decisions in patients with potentially avoidable infections<br>*(i.e. E.coli bacteraemia).*				
4.4	Does the AMT provide feedback and advice to prescribers who prescribe antimicrobials outside of the local guidelines when it is not justified?				
4.5	Dispensing of antimicrobials. Does the dispensary:<br>a. issue full packs of AMs<br>b. Issue the exact course length needed?<br>c. Issue Prepacks				

Originally developed by East of England Antimicrobial Pharmacy Network, 2016
Updated by PHE English Surveillance for Antimicrobial Utilisation and Resistance Group, 2020

5. Patient and Carers

No.	Criteria		Self-assessment	Evidence Provided	Reviewers Assessment
5.1	Is there a policy for providing information on AMs to patients?				
5.2	Is the trust able to demonstrate if patient are adequately informed about antibiotics (why they are prescribed, side effects and course of length)				
5.3	Is there clear information about the AMs the patient has received on the discharge letter including instructions on total course length and indication of antibiotic?				
5.4	According to NICE does the trust ensure the following information is provided when a patient care is transferred to another care setting<br>a. *Information on recent use of antibiotics*<br>b. *When the course should be reviewed*<br>c. *Information on who the patient should contact if they have concerns about infection*				

Originally developed by East of England Antimicrobial Pharmacy Network, 2016
Updated by PHE English Surveillance for Antimicrobial Utilisation and Resistance Group, 2020

6. Education and Training on the use of Antimicrobial

No.	Criteria		Self-assessment	Evidence Provided	Reviewers Assessment
6.1	Is there an AM education and training strategy?				
6.2	Is there mandatory core training in prudent antibiotic use for healthcare professionals at induction and at least every year. This should be repeated every 3 years and specifically cover those antibiotics that are linked to CDI.<br>*a. Doctors*<br>*b. Pharmacist*<br>c. *Nurses*				
6.3	Has the national Antimicrobial prescribing and stewardship competencies being used for the development of training for prescribers and other healthcare professionals?				

Details of Innovative Practice Not Included in the Criteria Above:

Recommendations to Improve AMS at the Host Site:

**Ward Visited:**
**Staff spoken to (please state profession and grade):**
**Questions asked:**<br><br>**Suggested questions to ask:**<br>Who is part of the AMT?<br>Do you receive regular feedback from antimicrobial audits (ask a prescriber)?<br>Can you show me where your antibiotic guidelines/policies are kept on the intranet?<br>Do you have an easily accessible printed summary of the antibiotic guideline?<br>Do you have the antibiotic guideline smartphone application on your phone?

Responses to questions:

Next steps:

It is recommended that the completed tool which includes both your self-assessment and peer review assessment alongside an action plan is shared with the Trust Antimicrobial Stewardship Committee, chief pharmacist and any other appropriate governance structure. You may also which to share this within the regional antimicrobial network so that learning can be distributed and an action plan to address any gaps can be agreed.

References

1. Pronovost PJ, Hudson DW. Improving healthcare quality through organisational peer-to-peer assessment: lessons from the nuclear power industry. BMJ Quality & Safety. 2012;21(10):872-5.
2. Roberts CM, Buckingham RJ, Stone RA, Lowe D, Pearson MG. The UK National Chronic Obstructive Pulmonary Disease Resources and Outcomes Project – a feasibility study of large-scale clinical service peer review. Journal of Evaluation in Clinical Practice. 2010;16(5):927-32.
3. O'Connor GT, Plume SK, Olmstead EM, et al. A regional intervention to improve the hospital mortality associated with coronary artery bypass graft surgery. JAMA. 1996;275(11):841-6.

Appendix 1 – Data Sheet

Comparison of hospital trust antimicrobial prescribing and resistance outcome data for the previous and most recent financial year *(where financial year data is unavailable compare by quarter).*

Hospital: ____________________ Date ____________________

	**Previous quarter**	**Most recent quarter**	**Data Source**
Four quarter rolling rate of total antibiotic prescribing **per 1000 admissions**; by acute trust			
Four quarter rolling rate of carbapenem prescribing **per 1000 admissions**; by acute trust and quarter			
Proportion of total antibiotic prescribing from the "Access" category of the WHO Essential Medicines List AWaRe index **(per cent)**			
Defined daily dose of antibiotics dispensed by Acute Trusts pharmacies to all inpatients and outpatients **per 1000 admissions**			
Defined daily dose of carbapenems dispensed by Acute Trusts pharmacies to all inpatients and outpatients **per 1000 admissions**			
Percentage of antibiotic prescriptions with evidence of review within 72 hours; by quarter			
C. difficile hospital-onset rates by reporting acute Trust and financial year			
E. coli bacteraemia hospital-onset counts and rates by NHS acute trust and financial year			
Percentage of frontline healthcare workers vaccinated with the seasonal influenza vaccine by NHS Acute Trust			

Appendix 2 –Antimicrobial Stewardship Peer Review Tool (Evidence Review Table)

No.	Criteria	Examples of evidence that can be provided	Policy/Standard that informed the criteria
1.1	Has the hospital management formally identified antimicrobial stewardship as a priority objective for the institution and included it in its key performance indicators?	Review with AMP<br>KIP report	NICE AMS guidance (1.1.2, 1.1.17)
1.2	Is there dedicated, sustainable and sufficient budgeted financial support for antimicrobial stewardship activities, training or IT support?<br>*(e.g. support for salary, information technology)*	Discuss with AMP	
1.3	Does the trust have a **<u>robust</u>** governance structure for the AMS programme/committee?<br>a. *Is there an Antimicrobial Stewardship committee?*<br>b. *Does the committee have a Terms of Reference?*<br>c. *Is there a MDT core membership with quorate list within the ToR?*<br>d. *Does the committee meet more than quarterly?*<br>e. *Is there a written AMS strategy/action plan that monitors adherence to good AMS prescribing principles in all clinical areas (at least annually)?*<br>f. *Actions are identified to address non-compliance with local guidance or unexpected trends in prescribing. These actions should be documented within the minutes of the ASC. Repeated non-compliance is formally investigated and is challenged by DIPC.*<br>g. *Are there clear lines (formal/written) of accountability to a higher committee e.g. Drug and Therapeutics Committee, Clinical Governance Committee or Infection Control Committee? Minutes*	ToR<br>Review minutes of ASC and audit reports<br>Strategy<br>Annual report	NICE AMS guidance (1.1.3) (1.1.4) (1.1.6) (1.1.8) (1.2.5); All ASC criteria is underpinned by the Code of Practice; Start Smart Then Focus; ASAT

	from ASC are discussed at this higher level meeting?<br><br>h. *Is a dedicated report produced at least annually which includes e.g. antibiotic use trends and/or prescription improvement initiatives, with time committed short term and long term measurable goals/targets for optimising antibiotic use?*		
1.4	Is there senior management support/responsibility for AMS?<br><br>*a. Does the Director of Infection Prevention and Control (DIPC) have AMS incorporated into their Job Description*<br><br>*b. Does the Trust/hospital have a senior manager responsible for AMS activities?*<br><br>c. *Is AMS addressed within the Trust Infection Prevention and Control (IPC) Strategy?*	Job Description for DIPC<br><br>IPC Strategy	ASAT
1.5	Who is part of the ASC? Has a whole health economy approach been adopted?<br><br>☐ Antimicrobial Pharmacist<br><br>☐ Microbiologist<br><br>☐ Infectious Diseases consultant<br><br>☐ Senior Nurse<br><br>☐ Paediatrician<br><br>☐ Primary Care Representative<br><br>☐ Acute Care Physician<br><br>☐ Senior member of the Pharmacy Management Team<br><br>☐ Anaesthetist<br><br>☐ Surgeon<br><br>☐ Director of Infection Prevention and Control<br><br>☐ Infection Prevention and Control Nurse<br><br>☐ Other Pharmacist	Review with AMP<br><br>TOR and previous ASC minutes<br><br>Evidence of attendance tracking?	Start Smart Then Focus; Code of Practice

	☐ Infection Prevention and Control Consultant<br><br>☐ CCG/CSU Pharmacist<br><br>☐ CCG Infection Prevention and Control Lead<br><br>☐ CCG commissioner<br><br>☐ Sepsis Lead Consultant<br><br>☐ Critical care outreach team nurse<br><br>☐ Lead Consultant for acute medicine<br><br>☐ Other (please specify below)		
1.6	Is there an Antimicrobial Pharmacist (AMP) identified as a leader for antimicrobial stewardship activities at your hospital?<br><br>a. *Does the AMP have written objectives and a PDP relating to the AMS strategy*<br><br>b. *Reviewing outpatient parenteral antibiotic therapy patients*<br><br>c. *The AMP plays an active part in local AMS networks across all care settings (i.e. local AMS networks, regional networks, UKCPA member)*<br><br>d. *Does the lead AMP have specialist training in infection (i.e. MSc in Infection Management) or greater than 3 years' experience within the specialist role?*<br><br>e. *Is the lead antimicrobial pharmacist working towards RPS faculty submission?*<br><br>f. *What whole time equivalent (WTE) AMP/500 beds are spent on AM duties?*	Review with AMP<br><br>Review personal development plan (PDP)<br><br>Minutes of regional network meetings<br><br>Attendance certificates from recent educational network meetings<br><br>RPS Faculty portfolio	ASAT; NICE AMS guidance (1.1.18)
2.1	Is there a robust AMS policy that clearly states the overall principles of AM use?<br><br>*(i.e. Antimicrobial prescribing policy which outlines responsibilities for staff)*	Policy	ASAT; Start Smart Then Focus; Code of Practice; NICE AMS guidance (1.1.4)
2.2	Does the policy clearly define roles, responsibility and procedures of the antimicrobial team and antimicrobial use?	Policy	Start Smart Then Focus; ASAT; Code of Practice; NICE AMS guidance

	a. initiation of AM within one hour for sepsis<br>*b. narrow spectrum, reviewing switching formulation of antibiotics*<br>*c. reviewing of microbiology results within 48-72 hours*<br>*d. prescribers documenting the review, use and switching of IV AMs*<br>e. *reminder for prescriber to consider resistant pathogens*		(1.1.16) (1.1.38) (1.1.32)
2.3	Does the policy or guidelines include the Start Smart then Focus treatment algorithm?	Review guidelines	Start Smart Then Focus
2.4	Do you have a robust method of sharing guidelines?	Review guidelines & policies	
2.5	Is there an up to date AM formulary?<br>*(i.e. a list of antimicrobials that have been approved for use in a hospital, specifying whether the drugs are unrestricted, restricted (approval of an antimicrobial stewardship team member is required) or permitted for specific conditions)*	Copy of guidelines and<br>Restricted List	Start Smart Then Focus; ASAT; NICE AMS guidance (1.1.9)
2.6	Is there a system for control of entry onto the formulary for new AMs and ongoing monitoring of costs, prescribing and patient outcomes?	Minutes of ASC or DTC	ASAT; NICE AMS guidance (1.2.1, 1.2.2, 1.2.4, 1.2.7, 1.2.8,1.2.9, 1.2.10)
2.7	Is selection for the guidelines informed by local sensitivity data?	Review guidelines with the AMP	ASAT; Start Smart Then Focus
2.8	Does your hospital have available and up-to-date recommendations (updated at least every year or when a new guideline is published) for AM guideline *(diagnosis, prevention and treatment)*, based on international/national evidence-based guidelines (such as NICE common infection guidelines) and local susceptibility (when possible), to assist with antimicrobial selection *(indication, agent, dose, route, duration)* for common clinical conditions, developed with the involvement of a clinical microbiologist or lead practitioner?	Review copy of UTI guideline, as an example and consider reviewing other guidelines<br>Minutes from DTC submission of guidelines	Start Smart Then Focus,<br>ASAT, Code of Practice, NICE AMS guidance (1.1.9) (1.1.24, 1.1.25, 1.1.27), Code of Practice

2.9	Has an MDT quality improvement programme for AMS been developed and is this sustained?	Copy of quality improvement programme	Start Smart Then Focus
2.10	Does your hospital support the antimicrobial stewardship activities/ strategy with adequate information technology services?<br><br>*(e.g. electronic summaries and guidelines available to wards, smartphone apps, decision support software)*	Review systems<br><br>Speak to ward staff	ASAT
2.11	Does the Microbiology lab use selective reporting of results in line with formulary choices?	Review systems	ASAT; NICE AMS guidance (1.1.22)
2.12	Are there timely microbiology results (including susceptibility data) preferably within 48 hours?	Review systems	Code of practice
2.13	Is susceptibility data (drug-bug combinations) and antimicrobial consumption reported to the national surveillance body?	Review PHE Fingertips AMR Portal	Code of practice
2.14	Is advice from a microbiologist/ID physician available by phone 24 hours a day?	Review with AMP	ASAT; Code of Practice
2.15	What ward rounds take place, what departments, frequency, and who attends?	Review with AMP & intervention data	ASAT; Start Smart Then Focus; NICE AMS guidance (1.1.10)
2.16	Is there evidence of audits/review that align with national AMR strategy/targets (e.g. CQUINs, 48-72hours reviews) and adherence to local guidelines?<br><br>a. Is the advice and data easily accessible?<br><br>b. Are the outcomes communicated to all staff in particular those who prescribe antibiotics?	Copy of audit reports<br><br>Review with AMP<br><br>Ask staff on ward	Start Smart Then Focus; NICE AMS guidance<br><br>(1.1.3, 2.1.1, 2.1.2); ASAT
2.17	Is evidence of an antimicrobial (AM) review/audit of specific antimicrobial agents or clinical conditions that can is easily accessed?<br><br>*(to be shared with the Trust board and other members of staff)*	Review audit strategy<br><br>Copy of Trust board report	ASAT; Start Smart Then Focus

Originally developed by East of England Antimicrobial Pharmacy Network, 2016
Updated by PHE English Surveillance for Antimicrobial Utilisation and Resistance Group, 2020

2.18	Is the information on AM prescribing feedback to prescribers?<br>*a. Is there regular feedback of audit data at a directorate level*<br>*b. Is there a system in place to feedback to prescribers patient safety incidents related to AM use and*<br>c. *Is there a system reporting lack of compliance to guidelines*	Review with AMP	NICE AMS guidance (1.1.6)
2.19	Are local systems and processes are in place for encouraging peer review of prescribing AMs?	Review with AMP	NICE AMS guidance (1.1.9, 1.1.19)
3.1	Does your hospital monitor and report antimicrobial consumption and prescribing data?<br>a. Broad spectrum antibiotics consumption<br>b. Local resistance patterns<br>c. Patient outcomes (e.g. LOS, mortality rates & readmissions)<br>d. Use national surveillance data for benchmarking<br>e. Measured ward level and consultant level	Review with AMP and reports	ASAT; NICE AMS guidance (1.1.13, 1.1.6, 1.1.15); Start Smart then Focus
4.1	Do you have the following guidance to improve patient safety?<br>*a. AM allergies guidance*<br>*b. IV to oral switch administration*<br>c. *Therapeutic Drug Monitoring guidance*	Review guideline	ASAT
4.2	Are incidents involving AMs reviewed at ASC meetings and incorporated into patient safety reporting systems to Boards and Commissioners? *(particularly hospital admissions for potentially avoidable life threatening infections, CDI and anaphylaxis)*	Review minutes and action plan	ASAT, Code of Practice, NICE AMS guidance (1.1.6)
4.3	Systems and processes are in place for identifying and reviewing whether hospital admissions are linked to previous	Review with AMP	NICE AMS guidance (1.1.7)

	prescribing decisions in patients with potentially avoidable infections<br>*(i.e. E.coli bacteraemia).*		
4.4	Does the AMT provide feedback and advice to prescribers who prescribe antimicrobials outside of the local guidelines when it is not justified?	Review with AMP	NICE AMS guidance (1.1.9), Start Smart Then Focus
4.5	Dispensing of antimicrobials. Does the dispensary:<br>a. issue full packs of AMs<br>b. Issue the exact course length needed?<br>c. Issue Prepacks	Review dispensary SOP with AMP	NICE AMS guidance (1.1.14)
5.1	Is there a policy for providing information on AMs to patients?	Review with policy	ASAT; NICE AMS guidance (1.1.31)
5.2	Is the trust able to demonstrate if patient are adequately informed about antibiotics (why they are prescribed, side effects and course of length)	Review with the AMP	ASAT; NICE AMS guidance (1.1.31)
5.3	Is there clear information about the AMs the patient has received on the discharge letter including instructions on total course length and indication of antibiotic?	Review with the AMP	ASAT; NICE AMS guidance (1.1.12, 1.1.31)
5.4	According to NICE does the trust ensure the following information is provided when a patient care is transferred to another care setting<br>*a. Information on recent use of antibiotics*<br>*b. When the course should be reviewed*<br>c. *Information on who the patient should contact if they have concerns about infection*	Review with the AMP	NICE
6.1	Is there an AM education and training strategy?	Review education and training strategy	ASAT
6.2	Is there mandatory core training in prudent antibiotic use for healthcare professionals at induction and at least every year. This should be repeated every 3 years and specifically cover those antibiotics that are linked to CDI.	Training slides	Start Smart Then Focus, Code of Practice, NICE AMS guidance (1.1.3, 1.1.10)

	a. Doctors<br>*b. Pharmacist*<br>c. *Nurses*		
6.3	Has the national Antimicrobial prescribing and stewardship competencies being used for the development of training for prescribers and other healthcare professionals?	Training slides	Start Smart Then Focus<br>NICE AMS guidance (1.1.10, 1.1.3)<br>HEE
